# Production of recombinant human transferrin using transgenic rice cell culture

**DOI:** 10.5511/plantbiotechnology.25.1105b

**Published:** 2026-03-25

**Authors:** Arisa Kubomura, Yuki Katayama, Yasuko Matsukura, Hayuma Otsuka, Toshiyuki Saeki, Kanako Sasaki, Ei-tora Yamamura, Hiroyuki Kajiura, Jong-kook Lee, Kazuhito Fujiyama, Hiroshi Okawa, Kazuaki Ohara

**Affiliations:** 1 Kirin Central Research Institute, Research & Development Division, Kirin Holdings Company, Ltd., 2-26-1 Muraoka-Higashi, Fujisawa, Kanagawa 251-8555, Japan; 2 International Center for Biotechnology, The University of Osaka, 2-1 Yamada-oka, Suita, Osaka 565-0871, Japan; 3 Department of Cardiovascular Regenerative Medicine and Drug Discovery, The University of Osaka Graduate School of Medicine, 2-2 Yamada-oka, Suita, Osaka 565-0871, Japan; 4 Department of Cardiovascular Medicine, The University of Osaka Graduate School of Medicine, 2-2 Yamada-oka, Suita, Osaka 565-0871, Japan

**Keywords:** *N*-glycan profile, recombinant protein, rice, transferrin

## Abstract

Transferrin is one of the major soluble serum proteins and is responsible for iron transport. Industrially, it is significant as a component of mammalian cell culture media, where a safe and stable supply is necessary. However, because transferrin is a glycoprotein containing 19 disulfide bonds, it is difficult to produce as a recombinant protein in bacteria, and at present it is mainly sourced from animals. In glycoprotein production, stability of the *N*-glycan profile is crucial, as glycans play important roles in diverse biological processes and influence the efficacy of glycoproteins. In this study, we aimed to produce recombinant human transferrin (rhTF) with stable *N*-glycan profiles. We generated transgenic rice calli expressing human TF (hTF) as a secretory glycosylated protein. rhTF was successfully produced as a soluble protein in the liquid culture medium of transgenic rice calli and subsequently purified. We confirmed that rhTF contained two plant-specific *N*-glycans and that these profiles were consistent across production batches. Purified rhTFs promoted the proliferation of cultured animal cells and human iPS cells, similar to serum-derived transferrin. Our results demonstrate new possibilities for producing recombinant glycoproteins with stable *N*-glycan profiles using a plant cell culture-based secretory protein expression system.

## Introduction

Recombinant protein production using plants has attracted considerable attention, and numerous proteins have been generated (Lee et al. 2023). Compared with mammalian-made pharmaceuticals, plant-made pharmaceuticals are more cost-effective to produce because plant cell cultures require only simple media and present a lower risk of contamination with mammalian pathogens or viruses (Buyel et al. 2017; Fukuzawa et al. 2024). Moreover, plants can perform post-translational modifications in a manner similar to mammals (Schillberg and Finnern 2021). In fact, in 2012 the U.S. Food and Drug Administration (FDA) approved β-glucocerebrosidase produced in carrot cells as an enzyme replacement therapy for type 1 Gaucher disease (Pastores et al. 2014; Shaaltiel et al. 2007).

Transferrin (TF) is a single-chain glycoprotein of approximately 77 kDa that contains two *N*-glycosylation sites and 19 disulfide bonds. TF is the major iron-binding protein in human plasma, responsible for the regulated delivery of iron to cells (Luck and Mason 2012). TF has long been used as an essential supplement in culture media for various mammalian cells due to the absolute requirement of iron for cellular growth and proliferation (Laskey et al. 1988). Although several reports have investigated the production of recombinant human transferrin (rhTF), successful expression of glycosylated rhTF in plants has not yet been demonstrated. Previously, rhTFs were produced in tobacco (*Nicotiana tabacum* cv. 81V9) leaves (Brandsma et al. 2010) and in rice (*Oryza sativa* L.) seeds (Zhang et al. 2010). Despite the conservation of amino acid sequences at the two *N*-glycan attachment sites, N432 and N630, the rhTFs produced in these studies lacked *N*-glycans.

In this study, we sought to produce rhTF with a stable *N*-glycan profile using plant cells. We demonstrated that transgenic rice calli could secrete rhTF with *N*-glycans at both N432 and N630, and that this rhTF exhibited mammalian cell proliferation activity equivalent to that of human TF (hTF) derived from human serum. Our findings indicate that transgenic rice calli provide a useful platform for producing glycoproteins with stable *N*-glycan profiles.

## Materials and methods

### Construction of transgenic rice calli expressing rhTF

**Figure 1:**
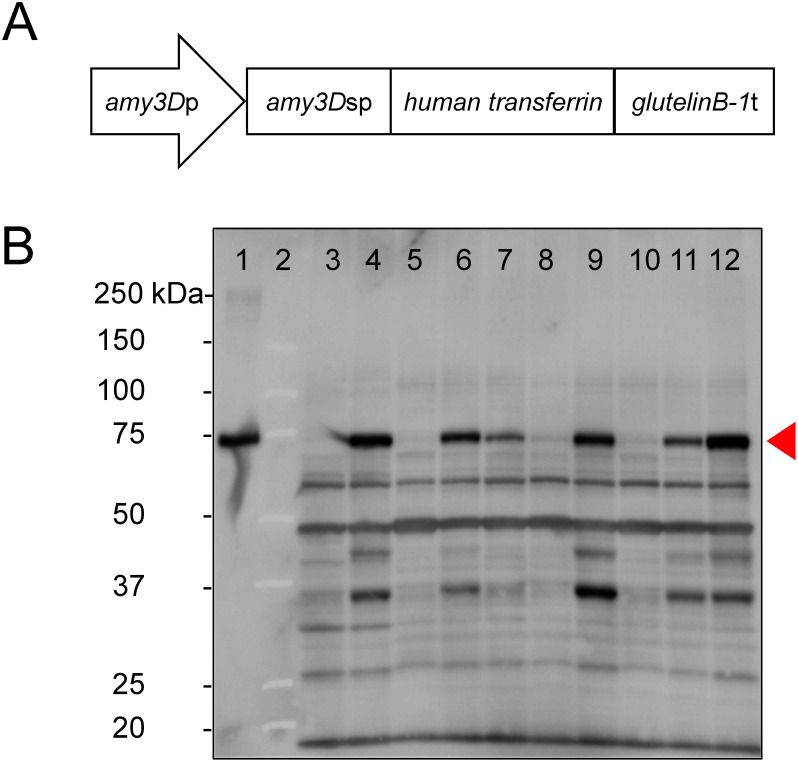
Figure 1. rhTF expression in transgenic rice calli. (A) Diagrammatic representation of the expression cassette for rhTF. *Amy3D*p: promoter of α-*amylase 3D* (GenBank Accession No. BAT05863); *amy3D*sp: signal peptide of α-*amylase 3D*; *human transferrin*: ORF of human transferrin (GenBank Accession No. NM_001063), lacking the signal sequence; *glutelinB-1*t: terminator of *glutelinB-1* (GenBank Accession No. AY585231). (B) Immunoblot analysis using anti-hTF. T_0_ calli were cultivated in N6D liquid medium without sucrose, and supernatants were collected on day 14. The samples were subjected to SDS-PAGE and immunoblotting with anti-hTF, followed by a peroxidase-conjugated anti-goat secondary antibody. The red triangle indicates bands corresponding to the glycosylated form of hTF. Lane 1: glycosylated TF from human serum (standard); Lane 2: size marker; Lane 3: culture supernatant of WT (negative control); Lanes 4–12: culture supernatants from T_0_ calli expressing rhTF.

The expression cassette consisted of rice seed germination α-*amylase 3D* promoter, a signal peptide of α-*amylase 3D*, the open reading frame of *hTF* without its native signal sequence, and the rice glutelin precursor *glutelinB-1* terminator (Figure 1A). The entire nucleotide sequence was synthesized with a *Sal*I restriction site at both the 5′ and 3′ ends of the cassette by Eurofins Genomics K.K. (Tokyo, Japan). The synthesized sequence was then inserted into pCAMBIA1300 (Abcam, Cambridge, England) using the *Sal*I site. The resulting plasmid was designated pCAMBIA1300-R3D_3D-SP_dTF_TgluB1. *O. sativa* cv. Nipponbare seeds were obtained from Nouken Co., Ltd. (Kyoto, Japan). Agrobacterium-mediated transformation and regeneration of transgenic T_1_ plants were performed as previously reported, with slight modifications (Nishimura et al. 2007). Transgenic rice calli were generated from T_1_ seeds and selected on hygromycin-containing R-N6D liquid medium. A transgenic T_1_ rice callus line was cryopreserved using a simple slow prefreezing method with an encapsulation technique, as described previously (Kobayashi et al. 2005).

### Expression of rhTF using transgenic rice cultured calli

For screening transgenic T_0_ callus lines, calli from each line were transferred into 200 ml of N6D (Nishimura et al. 2007) liquid medium containing 50 mg l^−1^ hygromycin and 500 mg l^−1^ carbenicillin disodium salt and cultured in the dark at 28°C and 80 rpm for 14 days. After 14 days of cultivation, the medium was removed and replaced with 200 ml of N6D liquid medium without sucrose, containing 50 mg l^−1^ hygromycin and 250 mg l^−1^ carbenicillin disodium salt, and the cultures were incubated in the dark at 28°C and 80 rpm for an additional 14 days.

For rhTF production using the selected T_1_ callus line, frozen calli were transferred into 200 ml of N6D liquid medium and cultured in the dark at 28°C and 80 rpm for 30 days. The resulting 200 ml of cell suspension culture was scaled up into 20 l of N6D liquid medium and cultured in the dark at 28°C and 89 rpm with aeration at 1 l min^−1^ for 20 days. The 20 l culture was further scaled up into 400 l of N6D liquid medium without sucrose and cultured in the dark at 32°C and 62 rpm with aeration at 5 l min^−1^ for 28 days.

### Purification of rhTF from transgenic rice cell culture supernatant

Purification of rhTF was performed as previously described with slight modifications (Zhang et al. 2010). The culture supernatant was filtered through a 0.45 µm depth filter (Pall, Port Washington, NY), followed by a 0.22 µm membrane filter (Pall). The clarified supernatant was concentrated tenfold using a 30 kDa regenerated cellulose membrane and diafiltrated against BF1 [50 mM NaCl and 10 mM sodium phosphate, pH 7.5]. The concentrated and diafiltrated sample was then purified by sequential immobilized metal-ion affinity chromatography (IMAC) and anion-exchange chromatography using an AKTA Pilot 600S system (Cytiva, Marlborough, MA). The sample was loaded onto an IMAC Fast Flow column (Cytiva) charged with Ni^2+^ and equilibrated with BF1. The column was washed sequentially with BF1, BF2 [1000 mM NaCl and 10 mM sodium phosphate, pH 7.5], and BF1 again to remove nonspecifically bound proteins. rhTF was eluted using a linear gradient of BF1 and BF3 [50 mM imidazole, 50 mM NaCl, and 10 mM sodium phosphate, pH 7.8]. The collected fraction was diluted fivefold with water and applied to a Capto Q ImpRes column (Cytiva) equilibrated with BF4 [25 mM Tris, pH 8.4]. rhTF was eluted using a linear gradient of BF4 and BF5 [500 mM NaCl and 25 mM Tris, pH 8.4]. The purified rhTF was diafiltrated against water and stored at 4°C.

### Expression analysis and quantification of rhTF

Secretory production of rhTF was confirmed by SDS-PAGE followed by immunoblotting. The culture supernatant was heated at 70°C for 10 min in sample buffer, separated on NuPAGE™ 4–12% Bis-Tris Protein Gels (Thermo Fisher Scientific, Waltham, MA), and transferred to polyvinylidene difluoride membranes (Thermo Fisher Scientific) using a semi-dry transfer method. After blocking with Blocking One (Nacalai Tesque, Kyoto, Japan) at 25°C for 1 h, the membranes were incubated for 1 h with Human Transferrin Antibody (Sigma, St. Louis, MO) at a 1 : 1000 (v/v) dilution, followed by incubation with peroxidase-conjugated AffiniPure Rabbit Anti-Goat lgG (Jackson ImmunoResearch, West Grove, PA). Protein band detection was carried out using ChemiLumi One (Nacalai Tesque) according to the manufacturer’s instructions. CBB staining was performed with Stain One Super (Ready to Use) (Nacalai Tesque). Quantification of rhTF was achieved by enzyme-linked immunosorbent assay (ELISA) using a Human Transferrin ELISA Kit (Bethyl Labs, Montgomery, TX) according to the manufacturer’s instructions.

### Gel filtration chromatography analysis of rhTF

Gel filtration chromatography analysis was performed using a Prominence HPLC system (Shimadzu, Kyoto, Japan). The flow rate and column temperature were maintained at 0.60 ml min^−1^ and 30°C, respectively. Twenty micrograms of rhTF was injected into a Shodex PROTEIN KW-803 column (i.d. 300×8.0 mm; Showa Denko, Tokyo, Japan) pre-equilibrated with buffer A [50 mM phosphate and 300 mM sodium sulfate, pH 7.0], and eluted in isocratic mode for 40 min with detection at 280 nm. For data analysis, peak annotation and peak area calculation were conducted using Labsolutions GPC software (Shimadzu).

### Liquid chromatograph-tandem mass spectrometry (LC-MS/MS) peptide map analysis of rhTF

LC-MS/MS of peptide samples was performed on a TripleTOF™ 5600 system (ABSciex, Framingham, MA) coupled with an Eksigent Ekspert™ nanoLC 415 (AB Sciex). One hundred ten micrograms of rhTF was mixed with iodoacetamide to a final concentration of 15 mM and incubated at room temperature for 15 min. The alkylated protein was desalted using an Amicon Ultra-0.5 3K device (Merck, Burlington, MA). Protease digestion was carried out with lysyl endopeptidase in the presence of ProteaseMax surfactant (Promega, Madison, WI) for 24 h at 37°C, after which trifluoroacetic acid (TFA) was added to a final concentration of 0.5% to inactivate residual protease. Chromatographic separation of the digested peptide mixture was performed by reverse-phase high-performance liquid chromatography (RP-HPLC) on an Eksigent column (i.d. 100×0.5 mm; ABSciex). The flow rate and column temperature were maintained at 5 µl min^−1^ and 40°C, respectively. For the mobile phase, peptides were injected into the column pre-equilibrated with 98% buffer A [0.1% formic acid in water] and 2% buffer B [0.1% formic acid in acetonitrile]. Separation was achieved using a linear gradient from 98 : 2 to 60 : 40 (buffer A : buffer B) over 50 min. Eluted fractions from RP-HPLC were introduced into the TripleTOF™ 5600 system (ABSciex). The mass spectrometer was operated in positive-ion mode with alternating precursor ion acquisition, using a collision energy of 10 eV and a full scan range of *m*/*z* 400–1250 for precursor ions. Spectra were acquired by time-of-flight analysis with a reflectron. Peptide identifications were mapped to chromatographic peaks using BioPharmaViewsoftware (AB Sciex).

### Glycosylation and *N*-glycan characterization of rhTF

Glycosylation and *N*-glycan analysis of rhTF were performed as previously described (Dent et al. 2022; Kajiura et al. 2022). Peptides detected by nanoLC-MS/MS were analyzed using DataAnalysis, BioTools, and Sequence Editor (Bruker Daltonics, Bremen, Germany).

### Cell proliferation activity assay of rhTF

The biological activity of rhTF was evaluated by its ability to support growth of the cultured hybridoma cell line AE1 (ATCC HB-72), as previously reported (Zhang et al. 2010) with slight modifications. AE1 cells were maintained in DMEM/F12 medium supplemented with insulin (10 µg ml^−1^), TF (5.5 µg ml^−1^), sodium selenite (0.0067 µg ml^−1^), ethanolamine (2.0 µg ml^−1^), and albumin (1 mg ml^−1^). For the proliferation assay, AE1 cells were washed with DMEM medium containing insulin (10 µg ml^−1^), sodium selenite (0.0067 µg ml^−1^), ethanolamine (2.0 µg ml^−1^), and albumin (1 mg ml^−1^), and seeded in 96-well plates at 4×10^3^ cells per well. rhTF and native holo-hTF (Sigma) were serially twofold diluted in DMEM medium, starting from a final concentration of 5 µg ml^−1^, and incubated with the cells under humidified conditions (37°C, 5% CO_2_) for 72 h. Cell proliferation was assessed using the WST-8 Cell Counting Kit (Dojindo Laboratories, Kumamoto, Japan). Twenty microliters of WST-8 reagent was added to each well, followed by incubation under humdified conditions (37°C, 5% CO_2_) for 4 h. Absorbance was measured at 450 nm and 650 nm using a SpectraMax M3 plate reader with SoftMax Pro software (Molecular Devices, San Jose, CA). The absorbance at 450 nm, corrected by subtracting the value at 650 nm, was used to plot concentration–response curves. The 50% maximal effective concentration (EC_50_) was calculated by regression to a four-parameter logistic model, and statistical analyses were performed by JMP software (SAS Institute, Cary, NC) and R software.

### iPS cell proliferation assay

The biological activity of rhTF was assessed by its ability to promote proliferation of cultured iPS cells (201B7). iPS cells were maintained in DMEM/F12 medium (Thermo Fisher Scientific) supplemented with L-ascorbic acid-2-phosphate magnesium (64 mg l^−1^), sodium selenite (14 µg l^−1^), FGF2 (100 µg l^−1^), insulin (19.4 mg l^−1^), NaHCO_3_ (543 mg l^−1^), TGFβ1 (2 µg l^−1^), and either rhTF or hTF (10.7 mg l^−1^) (Chen et al. 2011), and seeded in six-well culture plates coated with iMatrix-511 at 4×10^4^ cells per well. TrypLE Select (Thermo Fisher Scientific) was used to dissociate iPS cells. The cells were incubated under humidified conditions (37°C, 5% CO_2_) and passaged three times. After 7 days of culture, iPS cells were observed unstained and cell numbers were quantified under a stereomicroscope. Statistical analysis was performed by R software.

## Results

### Generation of rhTF-expressing rice callus lines

To produce rhTF using rice calli, the binary plant expression vector pCAMBIA1300-R3D_3D-SP_dTF_TgluB1, expressing rhTF under the control of the sugar starvation α-*amylase 3D* promoter, was constructed (Figure 1A). The α-amylase 3D signal peptide was fused to the N-terminal end of the mature hTF peptide to facilitate secretion of the recombinant TF. From Agrobacterium-mediated transformation of rice cells, 26 T_0_ callus lines were obtained. These lines were cultivated in N6D liquid medium (Nishimura et al. 2007), and rhTF production was evaluated from culture supernatants by SDS-PAGE. As shown in Figure 1B, a specific band at 75 kDa, the expected size for glycosylated hTF, was detected by immunoblotting with an anti-hTF antibody in the culture supernatants of transgenic T_0_ calli. No such signal was observed in the supernatant of wild type (WT) calli. The transgenic T_0_ callus line expressing the highest level of rhTF (lane 12, Figure 1B) was regenerated into a T_0_ plantlet. Forty-two seeds were harvested from this T_0_ plantlet, all of which generated T_1_ calli. One T_1_ callus line producing the highest level of rhTF was cryopreserved using a simple slow prefreezing method and employed in subsequent experiments.

### Purification and *N*-glycan structure of rhTF

**Figure 2:**
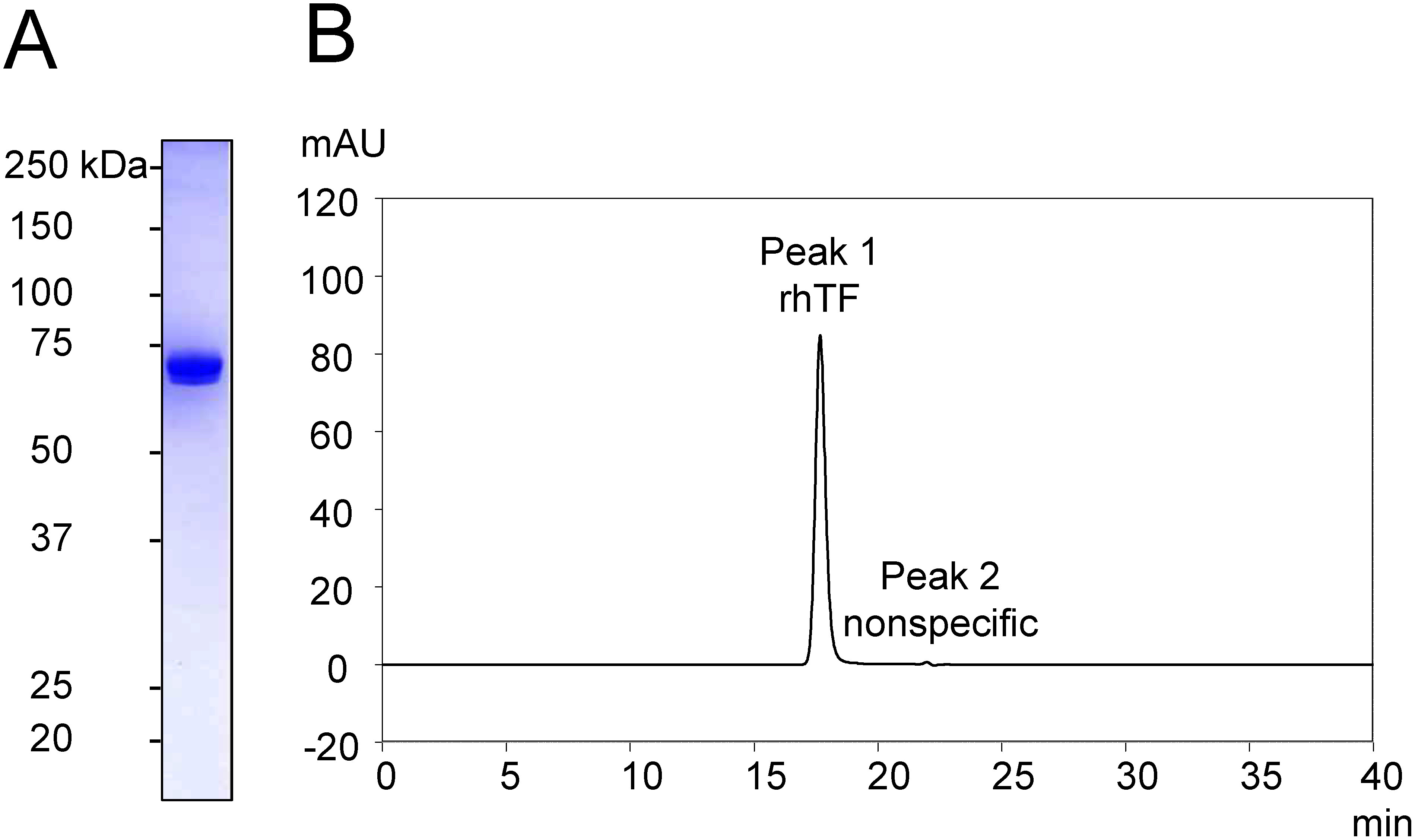
Figure 2. CBB staining and gel filtration chromatogram of purified rhTF. (A) CBB staining of purified rhTF. One µg of rhTF was applied. (B) Twenty µl of 1.0 mg ml^−1^ rhTF dissolved in Milli-Q water was injected. Peak 1, corresponding to hTF, represents rhTF, while Peak 2 is a nonspecific peak.

The amount of secreted rhTF in the supernatant was quantified by indirect ELISA, confirming production levels of 97 mg l^−1^. The rhTF was purified by a two-step chromatographic process consisting of immobilized metal-ion affinity chromatography and anion-exchange chromatography. Purity was assessed by SDS-PAGE and gel filtration chromatography. CBB staining of SDS-PAGE gels showed a nearly single band corresponding to rhTF (Figure 2A). Gel filtration chromatograms demonstrated that rhTF was eluted as a single peak, with no evidence of degradation products, polymers, or aggregates, and purity was determined to be greater than 99.5% based on peak area (Figure 2B, Supplementary Table S1). The overall purification yield of rhTF was 8.4% through the two-step chromatographic process.

To confirm the disulfide bond structure of rhTF, trypsin-digested rhTF peptides were subjected to LC-MS/MS, and the results were compared with the theoretical monoisotopic mass. This analysis showed values identical to the theoretical monoisotopic mass expected for disulfide bond-containing peptides (Table 1). These findings indicate that all 19 disulfide bonds were correctly formed in rhTF, consistent with the previously reported structure of hTF (Supplementary Figure S1).

**Table 1:** Table 1. List of disulfide bonds in rhTF identified by LC-MS/MS peptide mapping.

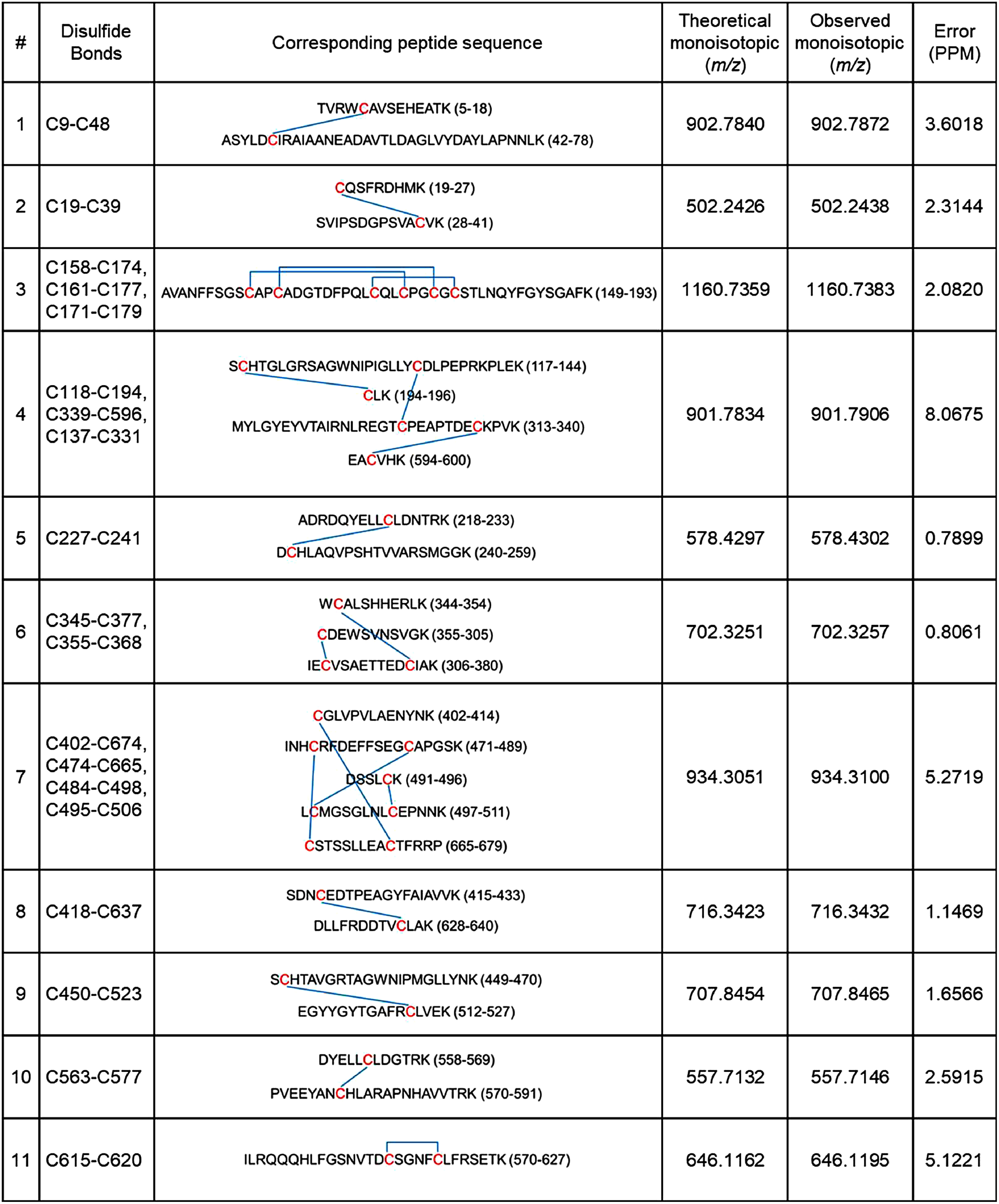

C residues shown in red indicate cysteines, and disulfide bonds are depicted by blue connecting lines.

### *N*-glycan profile of rhTF

**Figure 3:**
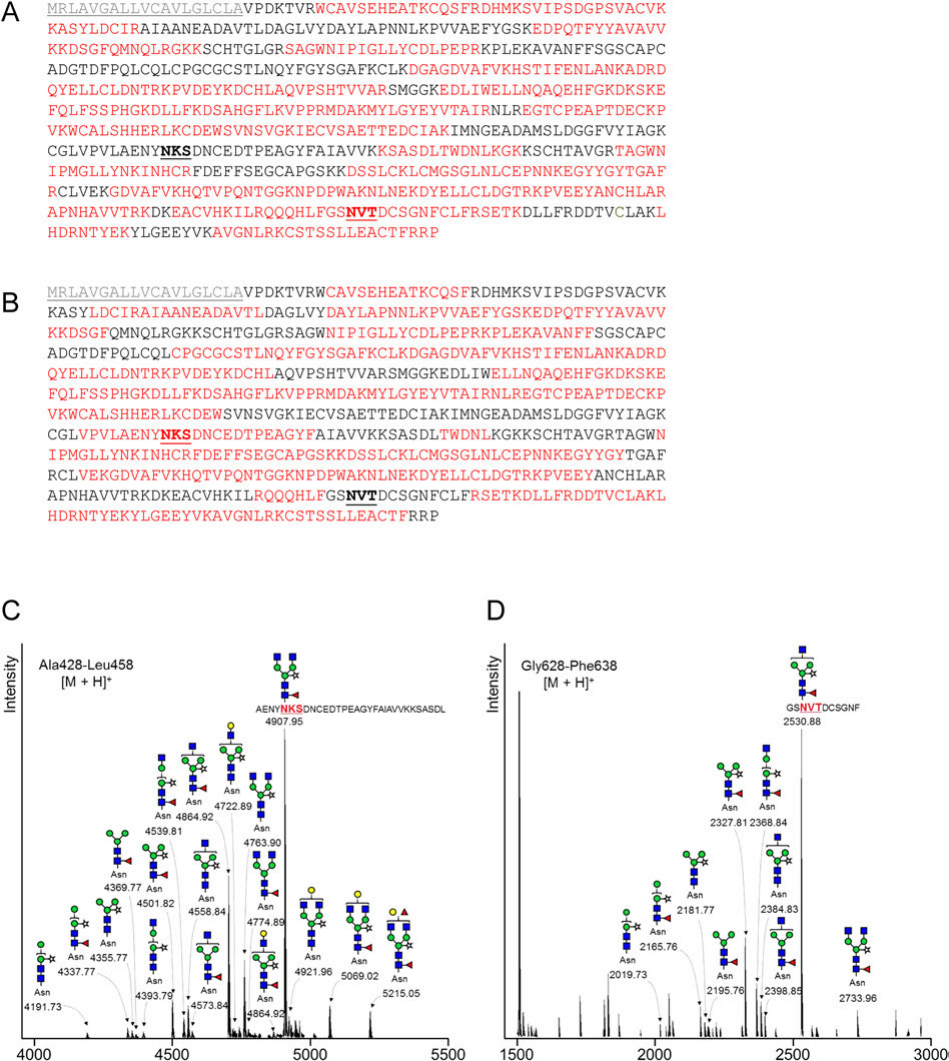
Figure 3. Glycosylation profile of rhTF. (A, B) Peptide mapping of purified rhTF digested with (A) trypsin or (B) chymotrypsin. The amino acid sequence of hTF is shown. Gray letters, red letters, and bold underlined letters represent the signal peptide, matched sequence, and conserved glycosylation site, respectively. (C, D) *N*-glycan structural analysis of (C) N432 and (D) N630 of purified rhTF. *N*-glycan structures on the glycopeptides were determined. Green circle: mannose; blue square: *N*-acetylglucosamine; red triangle: fucose; light yellow star: xylose; light yellow circle: galactose.

To examine the *N*-glycan profile of purified rhTF, structural analysis of *N*-glycans was performed. hTF has two conserved glycosylation sites, N432 and N630 (Supplementary Figure S1). Peptide mapping of protease-digested rhTF demonstrated that both N432 and N630 were glycosylated (Figure 3A, B). Glycosylation analysis at these sites was further performed using nanoLC-MS/MS, which revealed a variety of *N*-glycan structures at both N432 and N630 (Figure 3C, D). Peaks of 2-aminoprydine (PA)-labeled *N*-glycans corresponding to glucose oligosaccharide units were detected by RP-HPLC (Supplementary Figure S2). PA-glycans were also observed in LC-MS/MS analysis, and their relative abundances were calculated from peak areas. The dominant *N*-glycans exhibited plant-specific structures containing β1,2-xylose and core α1,3-fucose residues: ^GN^M3FX (39.8%), GN2M3FX (23.6%), and M3FX (17.0%) (Table 2).

**Table 2:** Table 2. Ratio of *N*-glycan structures.

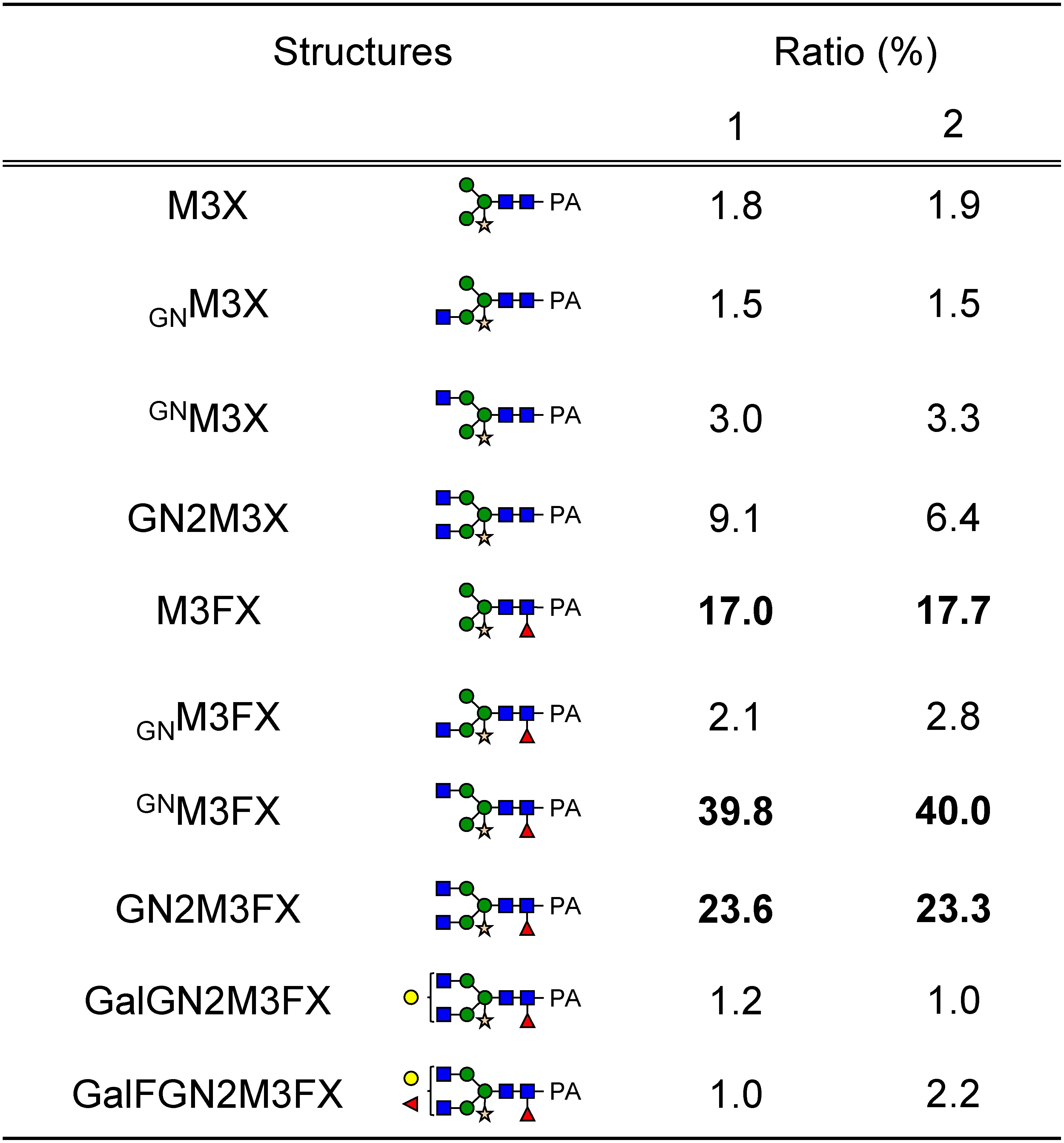

2-Aminopyridine (PA)-labeled *N*-glycans were analyzed by RP-HPLC. Collected peaks were further subjected to LC-MS/MS analysis, and ratios were calculated based on individual peak areas. Bolded ratios indicate dominant *N*-glycan structures. M (green circle): mannose; GN (blue square): *N*-acetylglucosamine; F (red triangle): fucose; X (light yellow star): xylose; Gal (light yellow circle): galactose.

The *N*-glycan profile of rhTF produced again under the same culture conditions was also examined. The dominant *N*-glycans were ^GN^M3FX (40.0%), GN2M3FX (23.3%), and M3FX (17.7%) (Table 2). These results indicate that the *N*-glycan profiles were stable across different production batches.

### Cell proliferation activity of rhTF

**Figure 4:**
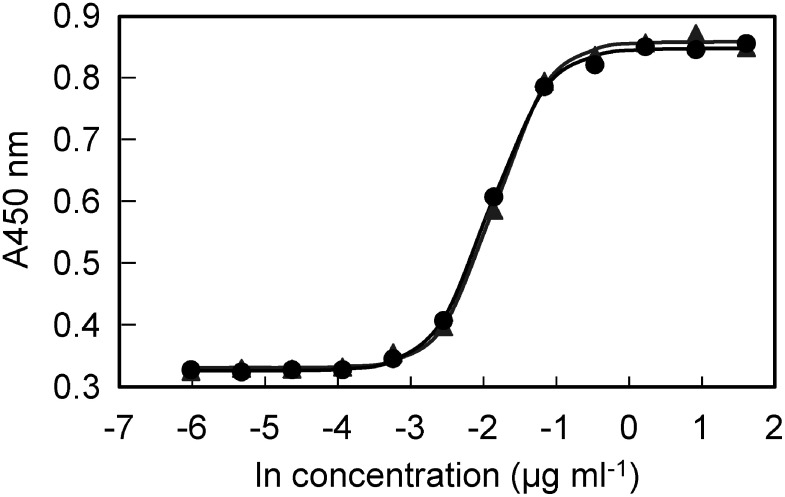
Figure 4. Proliferative response of the mouse hybridoma cell line AE1 to rice-derived purified rhTF and plasma-derived hTF. AE1 cells were incubated with increasing concentrations of purified rhTF or hTF for 72 h. Following incubation, tetrazolium salt was added as a chromogenic substrate, and the number of viable cells was determined by measuring the absorbance of the generated formazan at 450 nm/650 nm. The results are representative of two experiments. Data for rhTF and hTF were plotted as black circles and gray triangles, respectively. Growth curves fitted to sigmoid functions for AE1 cells treated with rhTF and hTF were drawn in black and gray, respectively.

To determine the biological activity of rhTF, we compared its cell growth–promoting activity with that of native holo-hTF using cultured hybridoma cells. For each sample, assays were performed in biological duplicates (*n*=2). rhTF or hTF was applied in twofold serial dilutions ranging from 5 µg ml^−1^ to 0.005 µg ml^−1^. Both proteins produced comparable dose-response curves for hybridoma cell proliferation (Figure 4). The A450 nm value in the absence of TF (negative control) was 0.328. Figure 4 shows that A450 values increased in a TF concentration-dependent manner. Saturated responses were observed at ∼0.6 µg ml^−1^ for both proteins, and the calculated 50% effective concentration (EC_50_) values for hTF was 0.20±0.01 µg ml^−1^, while that for rhTF was 0.17±0.01. According to the Wald test, the Wald statistic (Z value) for the difference in EC_50_ values between the two groups was 1.80, with a corresponding *p*-value of 0.07 (two-sided). Thus, there was no statistically significant difference in EC_50_ values between the two groups. These results indicate that rhTF exerted a proliferative effect on hybridoma cells comparable to that of native holo-hTF under serum-free conditions.

### iPS cell proliferation activity of rhTF

**Figure 5:**
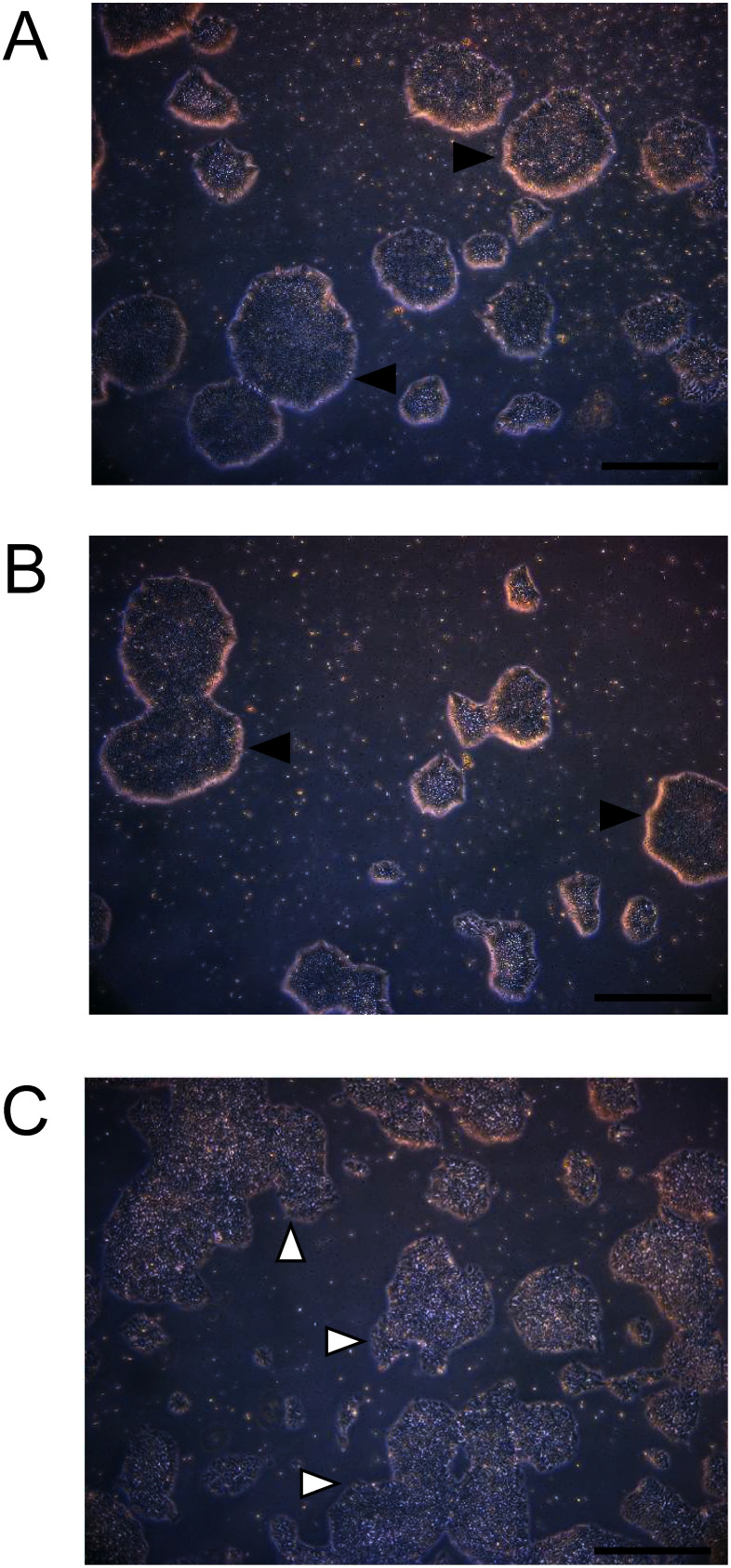
Figure 5. Proliferative response of iPS cells to TF. iPS cells were incubated with (A) rhTF, (B) hTF, or (C) without TF for seven days. Colonies were observed unstained using stereomicroscopic imaging. Scale bars represent 300 µm. Dense colonies are indicated by black triangles, and rough-edged colonies by white triangles.

The ability of rhTF to support iPS cell proliferation was also evaluated by supplementing purified rhTF to the iPS cell culture system. Regarding cell proliferation, iPS cells exhibited comparable growth in both TF-supplemented and TF-free media. After seven days of culture, cell proliferation relative to rhTF was 83.3±23.2% for hTF and 88.4±13.3% for the condition without TF by measuring cell numbers (*n*=3, no statistically significant difference, one-way ANOVA). In contrast, morphological observation revealed distinct differences among the culture conditions. Cells cultured with rhTF formed numerous dense colonies with no evidence of differentiation (Figure 5A), This morphology was consistent with that observed in cells cultured with hTF (Figure 5B). However, cultures in TF-free medium showed smaller, rough-edged colonies (Figure 5C). These results indicated that rhTF is functionally equivalent to hTF in sustaining the quality of iPS cell cultures.

## Discussion

In this study, we successfully produced rhTF using a rice suspension cell culture system. All 19 disulfide bonds in rhTF were properly formed, and plant-specific *N*-glycans were attached at both glycosylation sites. rhTF exhibited mammalian cell proliferation activity, and supplementation with rhTF in serum-free medium maintained human iPS cells in an undifferentiated state, equivalent to hTF.

For glycosylation, recombinant proteins must be processed through the endoplasmic reticulum, where the initial glycosylation steps conserved in eukaryotes occur (Schoberer and Strasser 2018), and through the Golgi apparatus, where *N*-glycans are modified into kingdom-specific structures (Strasser 2016). Because rhTF contains plant-specific *N*-glycans, it is presumed that the protein was correctly modified in the endoplasmic reticulum and Golgi apparatus of cultured rice cells. In this study, the α-amylase 3D signal peptide was used instead of the hTF-derived signal peptide. By contrast, previous studies reported that rhTF did not contain plant-specific *N*-glycans. For example, Brandsma et al. (2010) expressed rhTF in tobacco leaves with the hTF-derived signal peptide, while Zhang et al. (2010) expressed rhTF in rice endosperm with a glutelin signal peptide without removing the hTF-derived signal peptide. These studies suggest that post-translational modification of recombinant TF in those systems did not involve the pathway leading to plant-specific *N*-glycan addition. Several reports describe relationships between signal peptides and glycosylation. For instance, the signal peptide of cytotoxic T-lymphocyte antigen 4 determines the efficiency of post-translational modifications, including glycosylation (Anjos et al. 2002). In *Saccharomyces cerevisiae*, glycosylation depends on the cleavage of signal peptides by signal peptidases, suggesting an interaction between signal peptidases and glycosyltransferases (Chen et al. 2001). Therefore, appropriate selection of signal peptides that direct proteins through the secretory pathway may be critical for ensuring proper glycosylation. Our secretory protein production system has the potential not only to produce proteins with plant-specific *N*-glycans but also to enable attachment of desired *N*-glycan structures by engineering the Golgi pathway.

Previous studies have shown that allergic reactions caused by differences between mammalian- and plant-specific *N*-glycan structures rarely occur when plant-derived glycoproteins are administered to patients (Rup et al. 2017). Recombinant glucocerebrosidase produced by plant cells under standardized culture conditions exhibited identical *N*-glycan profiles across three batches and has been approved by the FDA as a pharmaceutical product (Shaaltiel et al. 2007). Similarly, clinical trials of virus-like particle influenza vaccines produced in *N. benthamiana* demonstrated good tolerability, with no major safety concerns associated with plant-type *N*-glycans (Pillet et al. 2019; Ward et al. 2014, 2020). Stabilization of the *N*-glycan profile may therefore contribute to ensuring the safety of glycoproteins, which is particularly critical for therapeutic applications. Controlling culture conditions—while accounting for changes in the culture environment due to host cell proliferation (e.g., fermentation heat)—appears to be important for stabilizing *N*-glycan profiles. In this study, the *N*-glycan profiles of rhTF produced in two independent batches under fixed culture conditions were identical (Table 2), indicating that our cultured rice callus–based recombinant protein production system exhibits sufficient stability to support consistent biomanufacturing and translational research applications.

Our study demonstrated the advantages of using cultured rice calli for recombinant protein production, particularly from the perspective of post-translational modifications. In the future, it may be possible to establish a platform technology that enables the production of more functional recombinant proteins by engineering *N*-glycans through molecular breeding of rice as a production host. In the case of carrot-derived glucocerebrosidase, the *N*-glycan structure remained stable in a maturely processed state (Shaaltiel et al. 2007), whereas the *N*-glycans of TFs produced in our rice calli system were stable in a pre-processed state, suggesting that the rice calli production system has favorable properties for *N*-glycan engineering. Therefore, the rice callus culture system developed in this study may also be applicable for the production of pharmaceuticals, particularly glycoproteins intended for clinical use. In conclusion, we successfully produced rhTF with correctly formed disulfide bonds and a stable *N*-glycan profile in rice calli. The rhTF exhibited similar activity to hTF in promoting cell proliferation and maintaining the undifferentiated state of iPS cells. Our study suggests that the secretory production system using rice calli may be applicable for the production of various complex proteins with appropriate glycosylation and biological activity.

## Abbreviations

ELISA: enzyme-linked immunosorbent assay
hTF: human transferrin
LC-MS/MS: liquid chromatograph-tandem mass spectrometry
PA: 2-aminoprydine
rhTF: recombinant human transferrin
RP-HPLC: reverse-phase high-performance liquid chromatography
TF: transferrin
WT: wild type

## Accession numbers

α-*amylase 3D*, GenBank Accession No. BAT05863; *hTF*, GenBank Accession No. NM_001063; *glutelinB-1*, GenBank Accession No. AY585231; *glutelin*, GenBank Accession No. Y00687.

## Description of supplementary files

Supplementary Figure S1. Amino acid sequence of hTF without signal peptide.

Supplementary Figure S2. Chromatogram of 2-PA labeled *N*-glycans analyzed by RP-HPLC.

Supplementary Table S1. Peak table of gel filtration chromatography of purified rhTF.
