# Supplementary material for: Production of recombinant human transferrin using transgenic rice cell culture
Source: Plant Biotechnol (Tokyo). 2026 Mar 25;43(1):73–81. doi: 10.5511/plantbiotechnology.25.1105b (PMC13170787; doi:10.5511/plantbiotechnology.25.1105b)
Supplement: Supplementary Data [file plantbiotechnology-43-1-25.1105b-s001.pdf]

Production of recombinant human transferrin using transgenic rice cell culture

A. Kubomura et al.

Supplementary files

Supplementary Figure S1. Amino acid sequence of hTF without the signal peptide.

Supplementary Figure S2. Chromatogram of 2-PA labeled *N*-glycans analyzed by RP-HPLC.

Supplementary Table S1. Peak table of gel filtration chromatography of purified rhTF.

Supplementary Figure S1

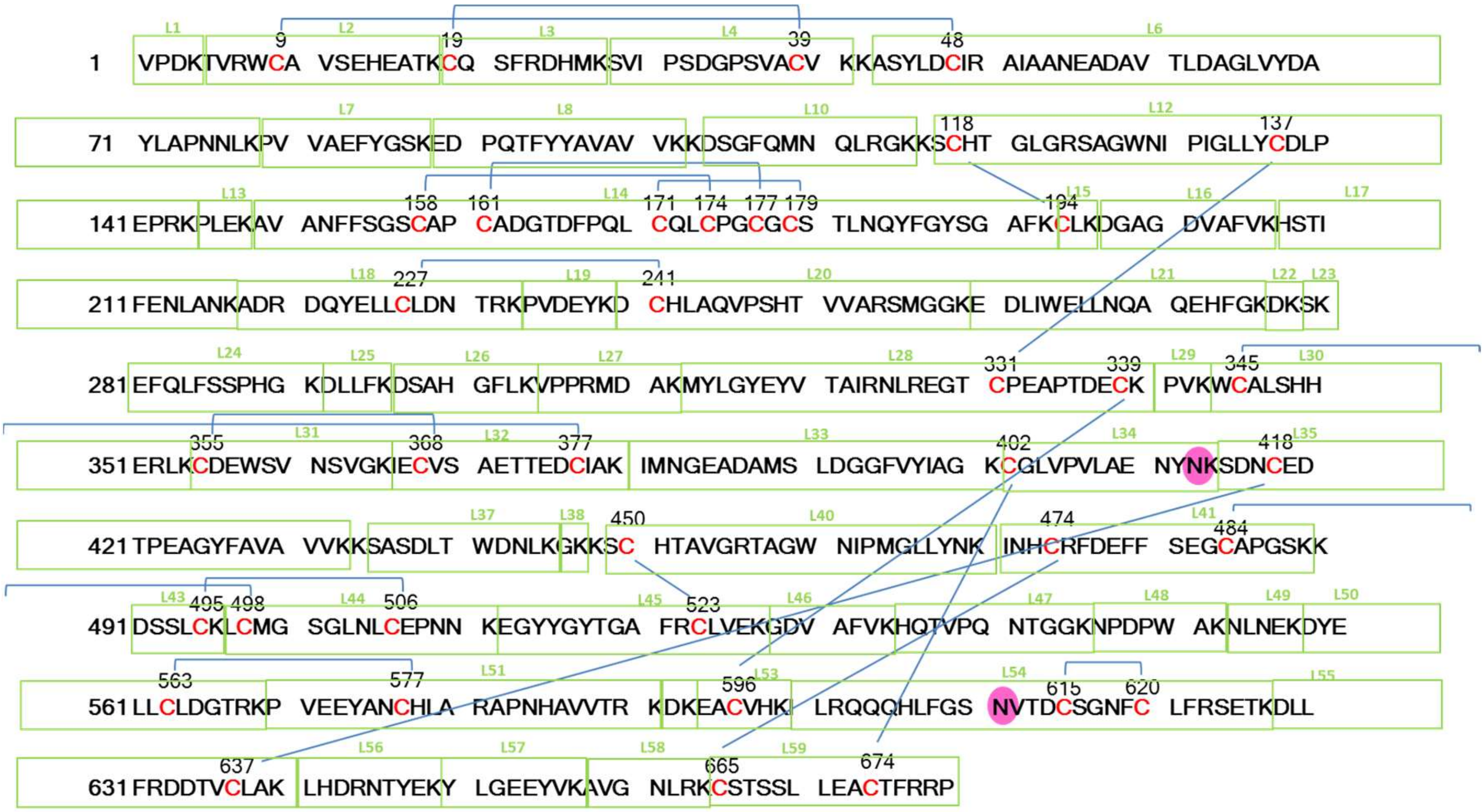

Supplementary Figure S1. Amino acid sequence of hTF (GenBank Accession No. NM_001063) without the signal peptide. Peptide sequences framed in green represent fragments generated by lysyl endopeptidase digestion. C residues in red indicate cysteines; blue lines show disulfide bonds; and N residues in pink represent conserved asparagine residues to which *N*-glycans are attached.

Supplementary Figure S2

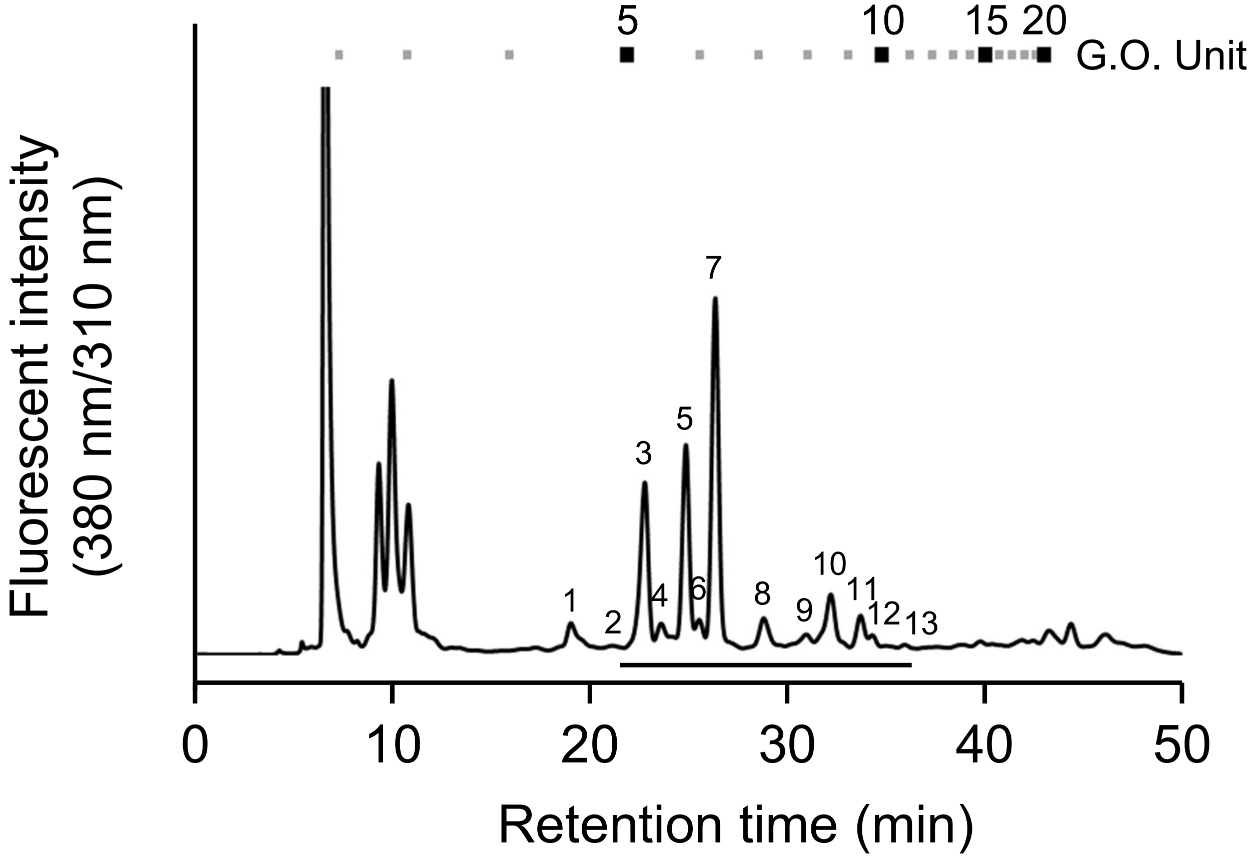

Supplementary Figure S2. Chromatogram of 2-PA-labeled *N*-glycans analyzed by RP-HPLC. Numbered peaks were collected and analyzed by LC-MS/MS.

Supplementary Table S1

Peak#	Compound	Retention time	Area	Area%
1	rhTF	17.667	2507182	99.50
2	(nonspecific)	21.983	12557	0.50

Supplementary Table S1. Peak table from gel filtration chromatography of purified rhTF. Peak annotation and area calculations were performed using LabSolutions GPC software (Shimadzu).
